# Upregulation of the Renin–Angiotensin System Pathways and SARS-CoV-2 Infection: The Rationale for the Administration of Zinc-Chelating Agents in COVID-19 Patients

**DOI:** 10.3390/cells10030506

**Published:** 2021-02-27

**Authors:** Loris Zamai

**Affiliations:** 1 Department of Biomolecular Sciences, University of Urbino Carlo Bo, 61029 Urbino, Italy; loris.zamai@uniurb.it; 2 National Institute for Nuclear Physics (INFN)-Gran Sasso National Laboratory (LNGS), Assergi, 67100 L’Aquila, Italy

**Keywords:** severe acute respiratory syndrome coronavirus-2, renin–angiotensin system, IL-10, convalescent plasma, albumin, zinc-chelating agents, vaccine, auto-immunity, MERS-CoV, ARDS

## Abstract

The article describes the rationale for the administration of zinc-chelating agents in COVID-19 patients. In a previous work I have highlighted that the binding of the SARS-CoV spike proteins to the zinc-metalloprotease ACE2 has been shown to induce ACE2 shedding by activating the zinc-metalloprotease ADAM17, which ultimately leads to systemic upregulation of ACE2 activity. Moreover, based on experimental models, it was also shown the detrimental effect of the excessive systemic activity of ACE2 through its downstream pathways, which leads to “clinical” manifestations resembling COVID-19. In this regard, strong upregulation of circulating ACE2 activity was recently reported in COVID-19 patients, thus supporting the previous hypothesis that COVID-19 may derive from upregulation of ACE2 activity. Based on this, a reasonable hypothesis of using inhibitors that curb the upregulation of both ACE2 and ADAM17 zinc-metalloprotease activities and consequent positive feedback-loops (initially triggered by SARS-CoV-2 and subsequently sustained independently on viral trigger) is proposed as therapy for COVID-19. In particular, zinc-chelating agents such as citrate and ethylenediaminetetraacetic acid (EDTA) alone or in combination are expected to act in protecting from COVID-19 at different levels thanks to their both anticoagulant properties and inhibitory activity on zinc-metalloproteases. Several arguments are presented in support of this hypothesis and based on the current knowledge of both beneficial/harmful effects and cost/effectiveness, the use of chelating agents in the prevention and therapy of COVID-19 is proposed. In this regard, clinical trials (currently absent) employing citrate/EDTA in COVID-19 are urgently needed in order to shed more light on the efficacy of zinc chelators against SARS-CoV-2 infection in vivo.

## 1. Introduction

The novel SARS-CoV-2 pandemic has led to a worldwide health and socioeconomic crisis. At present, despite numerous investments and scientific reports, hyperimmune plasma (a hundred-year-old practice) is the only SARS-CoV-2-specific therapy that is routinely used in clinical practice, although a few neutralising mAbs specific to SARS-CoV-2 antigens are now in clinical trials [1,2]. For severely affected patients not responding to chloroquine, antiviral, anti-inflammatory and anticoagulation combination therapies, hyperimmune plasma represents the last option despite some drawbacks [3,4,5]. High expectations and hopes are placed on SARS-CoV-2 vaccines. However, assessment of efficacy (duration of protection and protection from infection/transmission) and of long-term safety (occurrence of adverse events) for new vaccines still remain to be exhaustively determined [2,6]. Complications can stem from the generation of SARS-CoV non-neutralising- and/or auto-antibodies leading in some cases to the antibody-dependent enhancement of disease [5]. For example, SARS-CoV inactivated immunisation induced severe asthma-like immunopathology not soon after vaccination, but following SARS-CoV (re)challenged [5]. This is one of the reasons why vaccines against SARS-CoV-1 were not developed. On the other hand, the titanic undertaking of a massive COVID-19 vaccination program to reach herd immunity will have to face several practical difficulties. Moreover, both the apparent short-lasting immunity and the emergence of vaccine-resistant mutants, together with the current “globalisation” of COVID-19 infection, require a timely and synchronised world action that will be not easy to achieve, if only for the organisation and investment needed. Whether global herd immunity by vaccination will be hardly achievable, yearly SARS-CoV-2 vaccinations (as for influenza) of people at risk of severe forms will be instead feasible to urgently protect this (minority) part of the population. In this regard, other authors and I have already highlighted that individuals with low probability to survive to SARS-CoV-2 infection are usually elderly with comorbidities correlating with pre-existing high levels of circulating ACE2 concentration/activity that could be used as a prognostic marker [3,7,8,9]. Priority for vaccination should be given to the more vulnerable group of the population (possibly individuals with high systemic ACE2 activity) for which the risk/benefit balance is more favourable. However, elderly with comorbidities are often only marginally included in clinical trials; therefore, the risk of side effects and the effectiveness of new vaccines are less known in this specific group.

In light of these considerations, it is important to continue to search for new pharmacological treatments for COVID-19.

## 2. Anti-SARS-CoV-2 Bismuth-Based Drugs

A recent report shows that bismuth-based drugs can work as potent anti-SARS-CoV-2 agents in both in vitro cell lines and in vivo golden Syrian hamster model [10]. The proposed mechanism for SARS-CoV-2 inhibition is the impairment of zinc-dependent SARS-CoV-2 helicase through Zn^2+^ displacement. Indeed, different bismuth-based drugs are able to inhibit SARS-CoV-2 helicase in vitro; however, their enzymatic inhibition does not always correlate with their anti-SARS-CoV-2 activity when tested on different cell lines [10]. Interestingly, ranitidine bismuth citrate shows anti-SARS-CoV-2 activity even before virus entry [10,11], which is consistent with its activity on (zinc-dependent) surface proteins. Therefore, bismuth-based drugs may affect not only (helicase) viral but also host proteins predisposing them to face the viral threat. In this regard, several promising antivirals fail in vivo, indicating the importance of virus–host-drug interactions as a whole in determining therapy outcome.

Bismuth compounds have been widely used for *Helicobacter pylori* treatment by exploiting their inhibitory activity on bacterial enzymes [10,12]; however, they also inhibit (eukaryotic) yeast alcohol dehydrogenase by affecting its zinc-binding sites [12]. Given that zinc is functionally necessary in highly conserved zinc-binding domains of several viral and host proteins, in vivo SARS-CoV-2 inhibition by bismuth citrate-based drugs might therefore depend on its action on both viral and host proteins. However, what could the therapeutic targets of zinc displacement be in the host?

**Figure 1:**
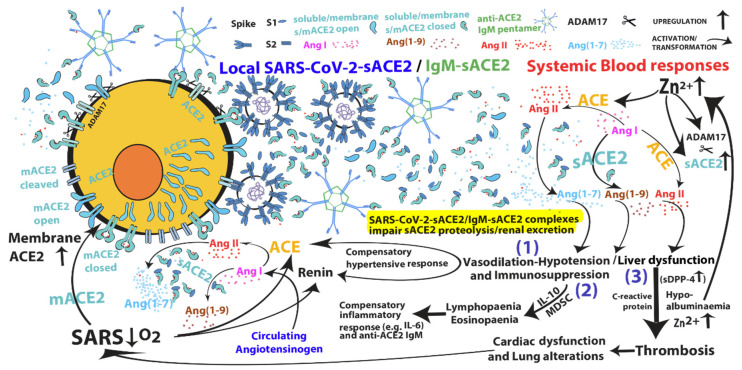
SARS-CoV-2 induced positive feedback loops mediated by RAS activation and pathophysiological consequences of the systemic excess of soluble ACE2 enzyme (sACE2). Upon spike protein binding to ACE2, ACE2 shedding leads to systemic upregulation of downstream pathways that can sustain positive feedback loops at different levels: (1) hypotensive effects mediated by Ang II catabolism can subsequently induce compensatory hypertensive response mediated by renin and ACE upregulation providing new Ang II for further Ang (1–7) production by ACE2; (2) Ang (1–7) antiproliferative and apoptotic effects, possibly through IL-10, may mediate eosinopaenia and lymphopaenia that impair immune system ability to counter virus infection and subsequently induce compensatory inflammatory responses; (3) liver dysfunction that, on one hand, increases plasma bioavailable Zn^2+^ (fuel for ACE, ACE2 and ADAM17 zinc-metalloprotease activities) due to reduced albumin (hypalbuminaemia) binding/sequestration of Zn^2+^, on the other hand, can produce procoagulant factors (e.g., C-reactive protein) leading to disseminated microvascular thrombosis, local hypoxic conditions (e.g., in heart and lungs) and consequent systemic hypoxia, which, in turn, upregulates renin, ACE and ACE2 syntheses and, therefore, peptide production of both arms of the RAS (i.e., Ang I, Ang II, Ang (1–7) and Ang (1–9)). Notably, upregulation of ACE2 synthesis increase membrane ACE2 (mACE2) expression that finally gives more chances to SARS-CoV-2 cell entry. Finally, both SARS-CoV-2 virions and anti-ACE2 non-neutralising IgM antibodies may increase ACE2 shedding and systemic activity, and sACE2-virion and sACE2-IgM complexes may impair the removal of systemic ACE2 enzymatic activity (for details see reference [3]).

I have already suggested that SARS-CoV-2 is able to induce shedding of the zinc-carboxypeptidase ACE2 by activating the zinc-metalloprotease ADAM17, finally leading to systemic upregulation of ACE2 activity in COVID-19 patients [3]. Based on this hypothesis, it is tempting to speculate that bismuth-based drugs may curb the upregulation of functional activities of both ACE2 and ADAM17 zinc-metalloproteases that are induced by SARS-CoV-2 infection. Actually, during SARS-CoV-1 infection, the ability of spike protein to reduce ACE2 surface expression suggested that the ACE2 pathway was down-modulated by ACE2 internalisation and that recombinant ACE2 could protect from severe lung failure [13,14]. Indeed, circulating (differently from membrane-bound) ACE2 is expected to protect from viral entry, and a pilot study of a clinical trial using recombinant ACE2 (rACE2) protein for COVID-19 started at the end of February 2020 by the Hospital of Guangzhou Medical University (ClinicalTrials.gov number, NCT04287686). Therapeutic activity of rACE2 was supposed to exert both inhibition of viral infection by masking the spike protein and reduction of Ang II excess, based on the assumption that SARS-CoV-2 induces the loss of ACE2 function [15,16,17]. Indeed, human rACE2 has been shown to inhibit SARS-CoV-2 infection in vitro in cell lines as well as in human organoids [18]. However, in vitro assays may not fully recapitulate the in vivo infection process. Unfortunately, the clinical trial assessing rACE2 in COVID-19 patients has been withdrawn without further details and the experimental data have produced only a case report of a single survivor of COVID-19, with no conclusive results [19]. Notably, several reports show that higher levels of ACE2 protein/activity are associated with advanced age, male sex, cardiopathies, hypertension, diabetes, dyslipidaemias and atherosclerotic plaques [7,8,20,21,22,23,24,25,26,27], which are also the main risk factors for COVID-19 complications. Therefore, it is not clear how COVID-19 comorbid patients having a high constitutive amount of circulating ACE2 may benefit from rACE2 administration, ultimately suggesting that the assumption that the ACE2 activity is down-regulated by SARS-CoV-2 may not be correct. In this regard, ACE2 surface down-modulation by SARS-CoV-1 has also been associated with ACE2 shedding produced by activation of ADAM17 zinc-metalloprotease [28,29]. Instead, NL63-CoV, a coronavirus that also binds to ACE2 with an affinity similar to that of SARS-CoV-1 [30], does not induce both severe respiratory symptoms and ADAM17-mediated ACE2 shedding [28,29], suggesting that ACE2 receptor cleavage may be crucial for SARS-CoV severity. In this regard, soluble forms of ACE2 (sACE2), induced or not by SARS-CoV binding, have been shown to retain not only their binding ability for spike viral proteins but also their enzymatic activity [28,31,32]. Therefore, the interaction of ACE2 with spike protein of SARS-CoVs would induce a cellular “protective” ACE2 shedding that limits viral entry but also an increase of systemic ACE2 activity. Compelling evidence to support this hypothesis comes from a recent article, which reports that circulating ACE2 activity in COVID-19 patients (at a median of 35-day post-infection) was 97-fold higher (*p* < 0.0001, and in accordance to disease severity) than control subjects, and it remained persistently elevated at 4-month post-infection [33]. This evidence strongly supports the above hypothesis, i.e., that the main targets of ranitidine bismuth citrate “in vivo” could be the zinc-metalloproteases that are upregulated during SARS-CoV-2 infection. Moreover, the confirmation that SARS-CoV-2 induces the upregulation of systemic ACE2 activity could also provide a rationale for the correlation between a high probability to develop severe forms of COVID-19 and a pre-existing high levels of circulating ACE2 that are typical of patient groups with comorbidities linked to severe COVID-19. In this regard, sACE2 and sACE2-spike protein complexes has been shown to maintain ACE2 enzymatic activity [28,31,32]; therefore, a high circulating ACE2 expression cannot be considered a mere disease biomarker. Rather, the dysregulated systemic function of ACE2 in some individuals would be further upregulated by SARS-CoV infections. Individuals with pre-existing high levels of circulating ACE2 activity would be predisposed to develop severe COVID-19 due to an excessive systemic ACE2 activity, which possibly leads to the worst COVID-19 outcomes. This could be the reason why systemic ACE2 protein/activity may work as a predictive biomarker for SARS development. However, is there any evidence of the detrimental effects of excessive ACE2 activity? In this regard, I have already reported the correlations between ACE2 pathway upregulation and clinical manifestations, such as immunosuppression, liver dysfunction, disseminated intravascular coagulopathy, cardiac dysfunction and respiratory syndrome, which would be sustained (after initial viral trigger) by different positive feedback loops involving, among others, IL-10, hypotension and local hypoxia followed by compensatory inflammatory cytokine secretion and hypertensive upregulation of renin and ACE zinc-metalloprotease, and ultimately severe acute respiratory syndrome (SARS) due to systemic hypoxia [3] (see Figure 1). Evidently, the potential roles of circulating elevated ACE2 activity in COVID-19 pathogenesis and putative treatments to inhibit its systemic activity warrant experimental validation, but certainly merit further attention.

## 3. Crucial Role of IL-10 in COVID-19 Pathogenesis

Among numerous cytokines upregulated in COVID-19, only IL-10 has tolerogenic, anti-inflammatory and anti-proliferative properties that, differently from pro-inflammatory cytokines, can be associated with eosinopaenia and lymphopaenia [3]; both early markers of COVID-19 that indicate an early IL-10 involvement in disease progression, possibly before a pro-inflammatory response [3]. Indeed, transcriptional responses to type I interferons (IFN-I) were reportedly low in severe COVID-19 as well as in SARS-CoV infected mice [34,35] despite COVID-19 patients display an early marked increase in plasma levels of IFN-α [36]. In this regard, IL-10 has been shown to partially exert its anti-inflammatory activities by inhibiting the transcription of IFN-inducible genes through suppression of STAT1 phosphorylation [37], suggesting that early production of IL-10 could mediate delayed inflammatory signalling, which has been suggested to contribute to a subsequent excessive inflammatory response [35]. Notably, chronic upregulation of IL-10 has already been shown to contribute to the progression of inflammatory diseases [38] [39,40], as it might occur in later/severe stages of COVID-19. In support of this hypothesis, IL-10-producing myeloid-derived suppressor cells (MDSCs) are massively and early expanded (at their first evaluation, 3/5-days after admission) in severe COVID-19 patients, which, at later recovery times, show both slow MDSC decline and concomitant inflammatory cytokine storm [41], ultimately indicating that inflammatory response may be elicited subsequently to immunosuppression (an immunosuppressive response followed by a counter-inflammatory process, finally leading to a form of “immune paralysis”) (see Figure 1). Indeed, longitudinal analyses to evaluate the dynamics of cytokine and chemokine production in plasma of COVID-19 patients reveal that in severe forms of COVID-19 there is an early systemic secretion (first week of onset of symptoms) of inhibitory mediators, including IL-10 and IL-1 receptor antagonist, whereas IL-6 and other inflammatory cytokines are significantly elevated only at later stages of severe COVID-19 illness [42]. The same report also shows that in COVID-19 patients there is an early upregulation of circulating levels of IFN-γ-inducible protein 10 (IP-10) [42]. Notably, IP-10, together with IL-6 and IL-10, is reported to constitute a severity-related triad that anticipates subsequent clinical progression (specifically) in COVID-19 patients, but not in non-COVID-19 patients with lower respiratory tract infections [36]. In this regard, administration of IL-10 in humans has been shown to enhance peripheral production of IP-10 and undesired pro-inflammatory effects [40], suggesting that IP-10 secretion is downstream IL-10. Finally, several lines of evidence suggest that the early and dramatic IL-10 elevation upon SARS-CoV-2 infection might play a detrimental pathological role in critically COVID-19 patients and IL-10 has also been proposed as a potential target for reducing COVID-19 mortality [3,39]. Indeed, IL-10 was strongly downregulated (*p* < 0.001, even more than IL-6, *p* < 0.01) in SARS-CoV-2-infected hamsters treated with ranitidine bismuth citrate [10], further suggesting its crucial role in disease pathogenesis.

## 4. Targeting of SARS-CoV-2-Upregulated Zinc-Dependent Pathways

To synthesise high amounts of functional SARS-CoV-2 helicase and ACE2, ADAM17 and ACE zinc-metalloproteases of renin-angiotensin system (RAS), cells of COVID-19 patients need high amounts of Zn^2+^. In this regard, the main zinc-transporter in plasma is albumin that, by binding Zn^2+^, reduces its bioavailability. Therefore, hypoalbuminaemia, typical of SARS-CoV-2 and other infections [3,43], can lead to an increase of bioavailable/functional and toxic forms of plasma Zn^2+^. However, the increase of bioavailable plasma Zn^2+^ can be easily imported into (endothelial) cells, finally allowing the upregulation of zinc-dependent cellular and viral functions eventually induced by SARS-CoV-2 infection, while reducing the total amount (concentration) of plasma Zn^2+^. Notably, the zinc status of an individual is usually assessed by plasma zinc concentration that both represents only 0.1% of the zinc in the body and widely depends on plasma zinc-transporters [3]. Therefore, particularly in the case of hypoalbuminaemia, plasma zinc concentration cannot be a reliable biomarker of cell/body zinc content, which is the zinc fraction playing a pivotal role in zinc-dependent functions and toxicity. In this regard, plasma zinc/albumin ratio could be considered as an alternative surrogate marker for functional zinc status [3]. Nevertheless, a (hypothetical) zinc deficiency is usually associated with low plasma concentration of zinc (<70 μg/dL), which are frequently detected transiently during infections or persistently in the elderly and in patients with chronic disease comorbidities that predispose to severe forms of COVID-19 [44,45,46,47,48]. Moreover, zinc ions have been shown to induce in vitro both inhibition of RNA virus replication and antiviral immune activity [44,45,46,47,48]. For these reasons, zinc supplementation has been proposed as an adjunct therapy for COVID-19, with several clinical trials underway [44,45,46,47,48] and a report indicating that zinc supplementation does not give protection from SARS-CoV-2 infection [49]. On the other hand, zinc displacement by ranitidine bismuth citrate has been shown to exhibit potent anti-SARS-CoV-2 activity in vivo [10], suggesting that reduction (rather than increase) of zinc-mediated activities may be effective against COVID-19. Indeed, elevated levels of zinc have been shown not only to stimulate pro-inflammatory cytokine secretion, but also inhibit allogeneic immune response (see [50]). In this regard, it is known that anthropic activities have caused soil and water contamination with heavy metals and zinc is one of the major toxic heavy metals that participate in environmental pollution [51,52]. Zinc concentrations in soil and water are rising due to industrial activities and the abnormal zinc concentrations can enter the food chain creating environmental stress for human beings [51]. For this reason, strategies for zinc removal from contaminated soil have been developed [51,52]. For example, the chelating agent ethylenediaminetetraacetic acid (EDTA) is one the most used in polluted soil treatments; however, its poor biodegradability has suggested the use of natural biodegradable chelants such as citric acid [52]. This evidence suggests that “hypernutrition” in developed countries could lead to excessive zinc absorption and its possible accumulation with age (rather than its deficiency). This can be true, particularly in sedentary people, which have a relatively low zinc excretion (via sweat and urine) [53,54]. Since sweat appears to be an important excretory pathway for zinc [53,54], it is possible that not only physical activity but also the increase of external temperatures as, for example, those related to seasonal (summer vs winter) changes or those induced by steam rooms, saunas or thermal treatments may favour zinc excretion, which together with temperature-induced vasodilation might affect the systemic activity of zinc-dependent RAS and consequently the predisposition to COVID-19.

Although zinc is considered a relatively nontoxic metal, excess zinc intake has been shown to contribute to cardiac dysfunction or amyloid beta-peptide plaque formation in Alzheimer’s disease [3,55]. Moreover, inhalation of welding fumes (mainly containing zinc) both increases pneumonia and cardiovascular risk and induces fever, fatigue, muscle ache, cough, dyspnoea, and a cytokine storm including IL-6 and IL-10, thus resembling the symptoms observed in COVID-19 patients [56,57]. Interestingly, inhalation of ZnCl_2_/ZnO/hexachloroethane (the main ingredients in smoke bombs) induces acute respiratory distress syndrome (ARDS) with clinical characteristics including lung radiographic and angiographic pictures that strongly resemble those of COVID-19 [50,58,59]. Since both ACE2 and ACE zinc-metalloproteases are activated by high concentrations of zinc (and chloride) ions [20,60], it is therefore possible to hypothesise that inhalation of fumes or chronic absorption/accumulation from contaminated food containing a high amount of zinc could induce the activation of both arms of the RAS (i.e., ACE and ACE2). An occurrence that seems to happen either in severe forms of COVID-19 or in comorbidities predisposing to it (for further details see [3]).

Notably, a structural work reveals that the full-length human ACE2 structure is assembled as a homodimer and molecular docking of spike protein trimers of SARS-CoVs onto the ACE2 homodimer suggests simultaneous binding of two spike protein trimers to an ACE2 homodimer on the plasma membrane [61]. The binding sites of spike protein are situated on S1 subunits and only a spike trimer with one S1 in an up conformation and two S1 in down conformations can bind to each ACE2 monomer, whose binding site is localised above its membrane-distal portion, far away from the catalytic site (see Figure 1). Indeed, the binding of soluble spike proteins to ACE2 receptors does not interfere with ACE2 both enzymatic activity and conformation (open/closed) changes [28,31,32,60,61]. However, although both ACE2 homodimers on the plasma membrane can associate with soluble spike proteins and soluble forms of ACE2 can bind spike proteins on virions independently on ACE2 open/closed conformations, it is plausible that two spike protein trimers placed on a virion can preferentially bind only to one of the two conformers of ACE2 homodimer anchored on the plasma membrane [61]. Indeed, a productive infection requires that SARS-CoV-2 virions dock onto the ACE2 homodimer placed on the plasma membrane with high binding affinity. In this regard, both structural analysis and computational approaches suggest that SARS-CoV-2 spike proteins tend to bind the closed/substrate-bound ACE2 conformer with higher affinity than the open one, which is likely due to differences in steric hindrance [61,62]. It is therefore possible that two spike proteins present on a virion preferentially bind the closed conformer of ACE2 homodimer when anchored on plasma membrane (see Figure 1). Since the closed ACE2 conformer (which seems preferential for virus binding/infection) needs the presence of both zinc and substrate in ACE2 catalytic site [60], zinc chelation or its displacement might also affect both ACE2 conformation and ACE2-mediated viral binding/entry. This eventuality could provide a reasonable explanation for the surprising observation that pre-treatment with bismuth-based drugs can confer cell resistance to SARS-CoV-2 infection even before the onset of viral replication [10].

These observations indicate that conformation, shedding and activity of zinc-metalloproteases upregulated by SARS-CoV-2 are expected to be particularly sensitive to reduction of plasma free Zn^2+^ concentrations. Notably, bismuth formulations usually contain metal chelating agents such as citrate, with which bismuth form stable complexes [10]. However, citrate chelates different cations and it is exploited in blood processing (e.g., plasmapheresis) as an anticoagulant Ca^2+^ chelator. Nevertheless, citrate (as EDTA) binds to Zn^2+^ at a remarkably higher affinity than Ca^2+^, and this ability has been shown to both bind to Zn^2+^ in catalytic site inhibiting carboxypeptidase A [63] and protect against zinc-induced intracellular cytotoxicity [64]. Notably, platelet Zn^2+^ release participates in procoagulant activities [65], suggesting a contribution of increased Zn^2+^ bioavailability to thrombotic predisposition in COVID-19; a phenomenon possibly countered by the anti-thrombotic activity of chloroquine [66], an enhancer of lysosome Zn^2+^ sequestration [67]. Therefore, anti-SARS-CoV-2 activity of bismuth citrate drugs may depend on both displacement of Zn^2+^ by Bi^3+^ and Zn^2+^/Ca^2+^ sequestration by citrate. Their combined action may involve various targets that affect a range of activities from Ca^2+^- and/or Zn^2+^-dependent coagulation to Zn^2+^-dependent enzymatic functions.

In general, the degree of zinc-dependent enzymatic inhibition by chelating agents is related to their both ability to accommodate into zinc-catalytic sites [63] and affinities for the cations that come into play (e.g., Zn^2+^ and Bi^3+^). For example, bismuth EDTA was a surprisingly poor inhibitor of SARS-CoV-2 helicase activity [11], indicating that both chelating drugs should not bind to bismuth too tightly and bismuth EDTA-based compounds cannot be effective in COVID-19.

Altogether, these observations suggest that plasma levels of bioavailable Zn^2+^, zinc-chelating agents, bismuth and albumin may affect zinc-dependent pathways upregulated by SARS-CoV-2 and, consequently, susceptibility to SARS-CoV-2 infection.

## 5. Efficacy and Limits of Convalescent Plasma Transfusion and Auto-Immune Complications

Convalescent plasma is a bio-complex that contains not only polyclonal antibodies against SARS-CoV-2, but also serum albumin, anticoagulants (e.g., citrate) and possibly other synergising or antagonising (e.g., C-reactive protein, ACE2 and SARS-CoV non-neutralising- or auto-antibodies) factors. Interestingly, passive immunisation has also been shown to predispose the immune system to actively counter infections. In this regard, exogenous antibodies of high affinity for pathogen antigens have been proposed to present antigens to B cells in germinal centres of lymphoid organs, finally allowing selection of B cells with even higher affinities [68]. Hyperimmune plasma is, therefore, a powerful antiviral tool that can act at multiple levels. However, its extensive use is limited by both the organisational complexity of selecting, collecting, storing and infusing convalescent plasma and latent risk of virus transfusion and/or of antibody-dependent enhancement of disease [5]. In this regard, a positive correlation between total antibodies against SARS-CoV-2 and clinical severity was observed late during infection (two weeks after illness onset) [69] and severe forms of COVID-19 were characterised by a surprising variety of auto-antibodies [70,71], suggesting that dysregulated humoral immunity contribute to COVID-19. In particular, neutralising (IgG) auto-antibodies against IFN-I, appear to contribute to severe forms of COVID-19 by antagonising innate antiviral responses [70,71], but also anti-ACE2 non-neutralising (IgM) auto-antibodies have been recently associated with severe clinical complications in some COVID-19 patients [72]. The IgM auto-antibody expansion is a condition that has been shown to correlate with upregulation of IL-6 (and IL-10) [73], suggesting that it might both be sustained by a compensatory inflammatory response and be inhibited by IL-6-targeted therapies, which could be more effective, for example, in specific cases of COVID-19 IgM expansion. Interestingly, anti-ACE2 IgM auto-antibodies have been shown to have no effect on ACE2 enzymatic activity, raising the possibility that anti-ACE2 IgM pentamers may mimic SARS-CoV-2 virion binding and membrane ACE2 release via ectodomain shedding (see Figure 1); an eventuality that could both maintain the systemic and deleterious ACE2 enzymatic activity even in the absence of viral load and provide a rationale for corticosteroid efficacy in COVID-19 [72]. Indeed, some late complications of COVID-19 that occur at low or undetectable levels of plasma viraemia and in the presence of anti-SARS-CoV-2 neutralising antibodies [72,74] might be explained by this hypothesis and might also suggest why later stages of COVID-19 are unaffected by convalescent plasma infusion [75]. Indeed, some convalescent plasma samples might contain anti-ACE2 auto-antibodies but, even more probably, may also have an elevated and detrimental ACE2 activity [33], an eventuality that should be considered when administrating convalescent plasma.

It is now emerging that SARS-CoV-2 infection leads to the appearance of various autoimmune and inflammatory diseases, whose molecular mechanisms are still unknown [76]. In this regard, I have recently described how the majority of protein structures can be “edited” by an intronic-mediated mechanism resembling that of immunoglobulin somatic hypermutation [77]. In particular, this occurs when genes are transcribed at their maximum rate (“hyper-transcribed”) and yet still are unable to meet environmental demands, i.e., their protein products are inadequate, and in this case, new genetic attempts can incidentally generate more adequate and functional proteins [77]. However, the majority of genetic attempts can generate dysfunctional proteins and/or proteins that can be recognised as extraneous (non-self) by the immune system, ultimately leading to autoimmunity [77]. It is tempting to speculate that, triggered by SARS-CoV-2 infection, hypertranscription of ACE2-downstream and immune-response pathways may induce the generation of several new gene attempts encoding non-functional proteins and/or new proteins which can be sensed as non-self by the immune system. In this regard, it has been shown that severe COVID-19 patients have either auto-antibodies that impact a wide range of immunological functions and, in particular, neutralising auto-antibodies against IFN-I or rare gene mutations predicted to be loss-of-function at 13 different loci that govern IFN-I immunity [70,71,78]. In this regard, despite early contradictory results regarding IFN-I response, there is increasing evidence of a robust IFN-I response in patients with severe COVID-19 [36,79,80], which is consistent with a hypertranscription of IFN-I genes and, following the above hypothesis, with the generation of non-functional IFN-I and anti-IFN-I auto-antibodies.

## 6. SARS-CoV-1/2 and MERS-CoV Severe Infections: Is It a Matter of the Liver?

Although triggered by distinct etiological factors, severe forms of SARS-CoVs, MERS-CoV and smoke-bomb-related acute respiratory distress (ARDS) syndromes share a common liver dysfunction [3,43,58,81,82,83]. Indeed, the elevation of hepatic procoagulant factors (e.g., C-reactive protein) and hypoalbuminemia are common underlying causes leading to thrombotic disease (distinct from disseminated intravascular coagulation [84]) and zinc toxicity, which may culminate in multi-organ dysfunction characterised by hypoxia and sudden worsening of disease-related symptoms (see Figure 1). Interestingly, circulating activity of dipeptidyl-peptidase-4 (DPP-4), the membrane receptor for MERS-CoV entry, is associated with chronic liver disease [85] and its shedding is induced by hypoxia and mediated by zinc-metalloproteases [86], thus sharing some features with ACE2 [3]. Indeed, DPP-4 also has widespread organ distribution, exists as a soluble circulating form in plasma and exerts pleiotropic effects via its peptidase activity [85]. The liver is one of the organs that expresses DPP-4 at high levels with a heterogeneous and specific lobular distribution; however, in chronic liver dysfunction, hepatic DPP-4 expression (and serum level) is further increased and DPP-4 inhibitors ameliorate hepatic injury [85]. Elevated serum DPP-4 activity also correlates with microalbuminuria unrelated to renal function [87]. Since constitutive DDP-4 presence in serum solutions mediates serum albumin cleavage (and release of an immunosuppressive dipeptide) [88], increased serum DPP-4 activity may affect albumin excretion and/or functions (e.g., zinc transport); an eventuality that should be evaluated before infusion of hyperimmune plasma and/or serum albumin solutions, and the co-administration of DPP-4 inhibitors might be considered. Notably, albuminuria was also found in some COVID-19 patients [89], suggesting that both SARS-CoVs and MERS-CoV may induce convergent pathological positive feedback loops involving both ACE2 and DPP-4 pathways (see Figure 1). In support of this hypothesis is the recent observational study showing that treatment with sitagliptin, a DPP-4 inhibitor, is associated with reduced mortality in COVID-19 patients [90]. If the hypothesis is correct, both SARS-CoVs and MERS-CoV infections but also some ARDS syndromes might be successfully treated with inhibitors of zinc-dependent pathways. Finally, it is tempting to speculate that receptor shedding induced by some anti-ACE2 (and possibly anti-DPP-4) non-neutralising (IgM) auto-antibodies might underlie some idiopathic forms of ARDS. Differently, anti-enzyme (IgG) neutralising auto-antibodies may generate other pathologic outcomes as, for example, asthma-like syndromes or some constrictive vasculopathies associated with pulmonary arterial hypertension and/or with persistent digital ischemia [91].

## 7. Conclusions

In a previous report, I had already suggested that zinc chelation might work against SARS-CoV-2 infection by acting on zinc-dependent RAS and coagulation pathways [3] and, from my perspective, the anti-SARS-CoV-2 activity of bismuth citrate-based drugs in vivo [10] is a “proof of concept” that the proposed hypothesis was correct. Therefore, bismuth-based drugs but also zinc-chelating agents (and exogenous albumin) might be potentially effective against COVID-19. We have potential therapeutic strategies by hindering (viral and host) zinc-dependent pathways that can be pursued in clinical trials or compassionate use protocols against COVID-19 and possibly other “zinc-dependent” diseases. The best compounds, administration routes and safety concerns have to be established. In this regard, oral administration of ranitidine bismuth citrate in humans has a well-documented safety profile; however, bismuth is poorly absorbed in the gastrointestinal tract, and the successful clinical outcome in vivo uses systemic (intraperitoneal) administration [10]. Unfortunately, systemic bismuth administration might be highly toxic and potentially unsafe for humans. Differently, citrate is an endogenous metabolite that (differently from EDTA) can easily both cross cell membranes and be metabolised, making it suitable for use in vivo. Indeed, citrate is promptly absorbed from the gastrointestinal tract and a small fraction is excreted in the urine [92]. Several studies evaluated the effectiveness of oral administration of alkali citrate (usually K and/or Mg) for preventive treatment of nephrolithiasis by exploiting its ability to sequester Ca^2+^ [92]. The daily administered dose of alkali citrate ranged from 3.5 to 10 g and lasted between 1.5 and 7 years [92]. Oral administration of alkali citrate through its renal excretion was shown to reduce the incidence of stone recurrences and, more important, the long-term treatments have no serious adverse effects and mainly limited to the gastrointestinal tract, which lead to a reduced treatment compliance [92]. However, zinc citrate supplementation has a good intestinal absorption [93], suggesting that the use of alkali citrate combined with food rich in zinc content might favour zinc intake. An alternative to citrate, whose therapeutic effect might not be lasting, is EDTA, a metal chelator widely used for zinc-metalloprotease inhibition [20]. Indeed, CaNa_2_EDTA, as well as other zinc-chelating agents and specific RAS pathway inhibitors (e.g., phytates, nicotianamine, zeolites, MLN-4760, Dx600, A779, aliskiren), have been already proposed as anti-SARS-CoV-2 agents whose administration routes and safety concerns have been widely discussed in a previous work [3]. Differently from citrate that can sequestrate both extracellular and intracellular Zn^2+^ [64], EDTA is an extracellular zinc chelator that is poorly absorbed by the gastrointestinal wall [3]. For this reason, it is preferentially administered by parenteral route; nevertheless, oral tablets of CaNa_2_EDTA can also work in reducing plasma zinc levels by inhibiting intestinal zinc absorption. On the other hand, the small adsorbed fraction diffuses mainly in the extracellular fluids, where it is not significantly metabolised but rapidly excreted by glomerular filtration [3]. Notably, the differences in bio-distribution (and affinity for zinc) of EDTA and citrate can be exploited in order to reduce body zinc availability. Indeed, the use of the two drugs in combination is expected to be more effective than alone. Citrate and EDTA are also well-known for their anticoagulant activity. In this regard, a recent report demonstrated the occurrence of thrombotic complications in a high number of COVID-19 patients despite high dosages of heparin, thus indicating the need to search for a better anti-thrombotic strategy [84,94]. Notably, zinc ion has been shown to promote clot stability by binding to fibrinogen and inhibiting heparin activity, possibly providing a rationale for heparin resistance [55]. It is therefore expected that combinations of heparin with zinc chelating agents may have a more effective anti-thrombotic activity than heparin alone, a possibility that should be tested in future studies.

Several aspects discussed in the present work suggest that chelating agents are expected to act in protecting from COVID-19 morbidity and mortality at different levels thanks to their both anticoagulant properties and inhibitory activity on zinc-metalloproteases. Indeed, the early onset of treatment with zinc chelating agents is expected to protect from the deleterious systemic ACE2 upregulation, which is early induced by SARS-CoV-2; this inhibitory activity will possibly reduce both the period of infection and the potential adverse effects of long-term treatments, hopefully rendering COVID-19 like a simple coronavirus cold, an immune eradicable disease. In this regard, oral supplementation of chelating agents is well-tolerated, safe, promptly available, easily deliverable and storable, inexpensive and practically usable also in developing countries. Chelating agent supplementation during the SARS-CoV-2 pandemic as an adjunct treatment to reduce the risk of infection and severe disease progression is therefore feasible and without problems of compliance. Based on the current knowledge of both beneficial/harmful effects and cost/effectiveness of chelating agents, it can be concluded that there is no reason to not initiate their use in the prevention and therapy of COVID-19. Unfortunately, clinical and preclinical data on this aspect are absent. Therefore, in order to shed more light on the efficacy of zinc chelators against SARS-CoV-2 infection in vivo, clinical trials employing citrate/EDTA in COVID-19 are urgently needed. Importantly, in order to achieve global protection and immunisation, preventive and therapeutic supplementation of chelating agents can be applied not only to developed but also to developing countries, where safe and cheap interventions are urgently needed.

Finally, I really hope that this work could be helpful to rightly direct our efforts against the pandemic and stopping the healthcare and socioeconomic disaster as soon as possible.

## Data Availability

No new data were created or analyzed in this study. Data sharing is not applicable to this article.
